# Dataset from dynamic shake-table testing of five full-scale single leaf and cavity URM walls subjected to out-of-plane two-way bending

**DOI:** 10.1016/j.dib.2019.103854

**Published:** 2019-03-20

**Authors:** U. Tomassetti, L. Grottoli, S. Sharma, F. Graziotti

**Affiliations:** a Dept. of Civil Engineering and Architecture – DICAr, University of Pavia and EUCENTRE, Pavia, Italy; b European Centre for Training and Research in Earthquake Engineering – EUCENTRE, Pavia, Italy; c UME School, Istituto Universitario di Studi Superiori – IUSS, Pavia, Italy; d DICAr, University of Pavia and EUCENTRE, Pavia, Italy

**Keywords:** Full-scale shaking table test, Two-way bending, Out-of-plane, Cavity wall, URM

## Abstract

This paper provides information related to the sensor measurements obtained from five different unreinforced masonry (URM) walls subjected to incremental dynamic shake-table tests at EUCENTRE, Pavia, Italy. This information has been made available to assist in the development and calibration of analytical and numerical models intended to simulate the out-of-plane (OOP) two-way bending response of URM walls. For further interpretation of the sensor recordings, and for a detailed discussion on the observed seismic performance of the specimens, the reader is referred to the article entitled “Experimental Response of URM Single Leaf and Cavity Walls in Out-Of-Plane Two-Way Bending Generated by Seismic Excitation” [1]. Videos documenting the failure of each specimen are also available on YouTube [2].

**Table:** Specifications table

Subject area	*Engineering*
More specific subject area	*Structural dynamics, Earthquake engineering*
Type of data	*Tables, figures, videos and recordings from instruments (acceleration, displacement and force time histories)*
How data was acquired	*The specimens were instrumented with accelerometers, wire potentiometers, linear potentiometers, and a three-dimensional motion-capture system was used for recording their response during testing.*
Data format	*Filtered and processed time histories: .txt files*
Experimental factors	*Specimens were U shaped: consisting of an out-of-plane panel and two return walls. Their materials can be considered representative of the URM building stock of the Groningen province of the Netherlands*
Experimental features	*Incremental unidirectional dynamic shake-table tests were performed up to near-collapse or collapse conditions of the specimens, using input ground motions compatible with induced-seismicity scenario for the Groningen region of the Netherlands*
Data source location	*The tests were carried out at the laboratory facilities of the European Centre for Training and Research in Earthquake Engineering (EUCENTRE) based in Pavia, Italy*
Data accessibility	*All recorded data (acceleration and displacement time histories) included with this article can also be requested on the EUCENTRE repository at the URL**www.eucentre.it/nam-project/?lang=en*
Related research article	Graziotti F, Tomassetti U, Sharma S, Grottoli L, Magenes G. Experimental response of URM single leaf and cavity walls in out-of-plane two-way bending generated by seismic excitation. Construction and Building Materials. 195, 2019, 650–670.; https://doi.org/10.1016/j.conbuildmat.2018.10.076[1]

**Table:** 

**Value of the data** • The data provides detailed information about the dynamic response of URM walls in two-way bending. It may serve as a benchmark for the development as well as calibration of numerical models to simulate the response of URM in the out-of-plane direction (e.g. Refs. [4], [5], [6]). • The data can also be used to validate simplified analytical methods to assess the response of URM in the out-of-plane direction. • The data may serve to evaluate the effectiveness of the test setup.

## 1 Data

Data corresponding to incremental dynamic testing of five full-scale URM walls is provided. Each specimen was densely instrumented with various sensors measuring accelerations and displacements throughout the testing sequence. The locations of these sensors and their operating status throughout the testing sequence (Table 4 of reference article [1]) is provided in Table 1, Table 2, Table 3, Table 4, Table 5 and Fig. 2, Fig. 3, Fig. 4, Fig. 5, Fig. 6. Fig. 2, Fig. 3, Fig. 4, Fig. 5, Fig. 6 also provide information on the mass distribution assumed to compute the provided inertial force associated with each specimen. The recorded data is organized into folders with each folder corresponding to a single specimen. Each folder contains several files with a single file containing the data of all instruments recording in a particular test. The name of each file provides information about which test of the testing sequence it contains data of. Within each .txt file, the first column corresponds to a time vector whereas all other columns correspond to instrument readings. All data acquired was filtered from frequencies higher than 50 Hz. All recordings of accelerations and forces are provided in units of g and kN, respectively while displacements are given in mm.

**Table 1:** CS-010/005-RR data organisation.

Col.	Instr.	Description	Offline	Location		Associated Mass		
				X [mm]	Z [mm]	1^st^ [kg]	2^nd^ [kg]	3^rd^ [kg]
1	–	‘Time [s]’	–	–	–	–	–	–
2	Acc.	‘Shake Table Acc. [g]’	–	–	–	–	–	–
3	Acc.	‘Foundation Acc. [g]’	–	–	–	449	449	284
4	Acc.	‘Frame Acc. [g]’	–	–	–	–	–	–
5	Acc.	‘Side A Beam Acc. [g]’	–	–	–	–	–	–
6	Acc.	‘Centre Beam Acc. [g]’	–	–	–	412	412	260
7	Acc.	‘ Side C Beam Acc. [g]’	–	–	–	–	–	–
8	Acc.	‘1/4 B Wall Acc. [g]’	–	1995	775	294	206	206
9	Acc.	‘1/2 A Wall Acc. [g]’	–	885	1425	229	228	402
10	Acc.	‘1/2 B Wall Acc. [g]’	–	1995	1425	163	137	136
11	Acc.	‘1/2 C Wall Acc. [g]’	–	3105	1425	229	357	531
12	Acc.	‘3/4 B Wall Acc. [g]’	–	1995	2070	281	268	267
13	Pot.	‘Shake Table Disp. [mm]’	–	–	–	–	–	–
14	WP	‘1/4 A Wall Disp. [mm]’	1, 9, 23, 27	885	775	–	–	–
15	WP	‘1/4 B Wall Disp. [mm]’	1, 9, 23, 27	1995	775	–	–	–
16	WP	‘1/4 C Wall Disp. [mm]’	1, 9, 23, 27	3105	775	–	–	–
17	WP	‘1/2 A Wall Disp. [mm]’	1, 9, 23, 27	885	1425	–	–	–
18	WP	‘1/2 B Wall Disp. [mm]’	1, 9, 23, 27	1995	1425	–	–	–
19	WP	‘1/2 C Wall Disp. [mm]’	1, 9, 23, 27	3105	1425	–	–	–
20	WP	‘3/4 A Wall Disp. [mm]’	1, 9, 23, 27	885	2070	–	–	–
21	WP	‘3/4 B Wall Disp. [mm]’	1, 9, 23, 27	1995	2070	–	–	–
22	WP	‘3/4 C Wall Disp. [mm]’	1, 9, 23, 27	3105	2070	–	–	–
23	Pot.	‘4/4 A Wall Disp. [mm]’	All Tests	–	–	–	–	–
24	Pot.	‘Top Beam Disp. [mm]’	–	–	–	–	–	–
25	Pot.	‘4/4 C Wall Disp. [mm]’	All Tests	–	–	–	–	–
26	Pot.	‘1/2 Side A OOP Detachment [mm]’	–	220	1425	–	–	–
27	Pot.	‘Side A Ret. Wall Sliding [mm]’	1–27	50	450	–	–	–
28	Pot.	‘1/2 Side C OOP Detachment [mm]’	–	3770	1425	–	–	–
29	Pot.	‘Side C Ret. Wall Sliding [mm]’	1–27	3935	450	–	–	–
30	–	‘Inertial Force [kN]’	1, 9, 23, 27	–	–	–	–	–

**Table 2:** CS-000-RF data organisation.

Col.	Instr.	Description	Offline	Location		Associated Mass	
				X [mm]	Z [mm]	1^st^ [kg]	2^nd^ [kg]
1	–	‘Time [s]’	–	–	–	–	–
2	Acc.	‘Shake Table Acc. [g]’	–	–	–	–	–
3	Acc.	‘Foundation Acc. [g]’	–	–	–	452	432
4	Acc.	‘Frame Acc. [g]’	–	–	–	–	–
5	Acc.	‘Side A Ret. Wall Acc. [g]’	–	–	–	103	
6	Acc.	‘Top Beam Acc. [g]’	All Tests	–	–	–	
7	Acc.	‘Side C Ret. Wall Acc. [g]’	–	–	–	103	
8	Acc.	‘1/4 B Wall Acc. [g]’	20–22	1995	615	294	–
9	Acc.	‘1/2 A Wall Acc. [g]’	20–22	885	1425	287	–
10	Acc.	‘1/2 B Wall Acc. [g]’	20–22	1995	1425	249	1625
11	Acc.	‘1/2 C Wall Acc. [g]’	20–22	3105	1425	287	–
12	Acc.	‘4/4 B Wall Acc. [g]’	–	1995	2070	281	–
13	Pot.	‘Shake Table Disp. [mm]’	–	–	–	–	–
14	Opt.	‘1/4 A Wall Disp. [mm]’	1, 5, 19-22	885	775	–	–
15	Opt.	‘1/4 B Wall Disp. [mm]’	1, 5, 19-22	1995	775	–	–
16	Opt.	‘1/4 C Wall Disp. [mm]’	1, 5, 19-22	3105	775	–	–
17	Opt.	‘1/2 A Wall Disp. [mm]’	1, 5, 19-22	885	1425	–	–
18	Pot./Opt.	‘1/2 B Wall Disp. [mm]’	1, 5, 18-22	1995	1425	–	–
19	Opt.	‘1/2 C Wall Disp. [mm]’	1, 5, 19-22	3105	1425	–	–
20	Opt.	‘3/4 A Wall Disp. [mm]’	1, 5, 19-22	885	2070	–	–
21	Opt.	‘3/4 B Wall Disp. [mm]’	1, 5, 19-22	1995	2070	–	–
22	Opt.	‘3/4 C Wall Disp. [mm]’	1, 5, 19-22	3105	2070	–	–
23	Opt.	‘4/4 A Wall Disp. [mm]’	19–22	885	2720	–	–
24	Pot.	‘4/4 B Wall Disp. [mm]’	1, 5, 19	1995	2720	–	–
25	Opt.	‘4/4 C Wall Disp. [mm]’	19–22	3105	2720	–	–
26	Pot./Opt.	‘1/2 Side A OOP Detachment [mm]’	19–22	105	1340	–	–
27	Pot./Opt.	‘4/4 Side A OOP Detachment [mm]’	19–22	105	2640	–	–
28	Pot./Opt.	‘1/2 Side C OOP Detachment [mm]’	19–22	3880	1340	–	–
29	Pot./Opt.	‘4/4 Side C OOP Detachment [mm]’	19–22	3880	2640	–	–
30	–	‘Inertial Force [kN]’	1, 5, 19	–	–	–	–

**Table 3:** CSW-000-RF data organisation.

Col.	Instr.	Description	Offline	Location		Associated Mass	
				X [mm]	Z [mm]	1^st^ [kg]	2^nd^ [kg]
1	–	‘Time [s]’	–	–	–	–	–
2	Acc.	‘Shake Table Acc. [g]’	–	–	–	–	–
3	Acc.	‘Foundation Acc. [g]’	–	–	–	369	417
4	Acc.	‘Frame Acc. [g]’	21–27	–	–	–	–
5	Acc.	‘Side A Ret. Wall Acc. [g]’	–	–	–	103	–
6	Acc.	‘Top Beam Acc. [g]’	All Tests	–	–	–	–
7	Acc.	‘Side C Ret. Wall Acc. [g]’	–	–	–	84	–
8	Acc.	‘1/4 B Wall Acc. [g]’	21–27	2325	615	72	–
9	Acc.	‘1/2 A Wall Acc. [g]’	–	885	1425	98	331
10	Acc.	‘1/2 B Wall Acc. [g]’	21–27	1330	1425	99	–
11	Acc.	‘1/2 C Wall Acc. [g]’	–	3380	1425	47	235
12	Acc.	‘4/4 B Wall Acc. [g]’	–	1975	2560	237	548
13	Pot.	‘Shake Table Disp. [mm]’	–	–	–	–	–
14	Opt.	‘1/4 A Wall Disp. [mm]’	1-2, 10, 21	665	775	–	–
15	WP/Opt.	‘1/4 B Wall Disp. [mm]’	1-2, 10, 21	1495	775	–	–
16	Opt.	‘1/4 C Wall Disp. [mm]’	1-2, 10, 21	3380	775	–	–
17	Opt.	‘1/2 A Wall Disp. [mm]’	1-2, 10, 21	665	1425	–	–
18	WP/Opt.	‘1/2 B Wall Disp. [mm]’	1-2, 10, 21	1495	1425	–	–
19	Opt.	‘1/2 C Wall Disp. [mm]’	1-2, 10, 21	3380	1425	–	–
20	Opt.	‘3/4 A Wall Disp. [mm]’	1-2, 10, 21	665	2315	–	–
21	WP/Opt.	‘3/4 B Wall Disp. [mm]’	1-2, 10, 21	1495	2315	–	–
22	Opt.	‘3/4 C Wall Disp. [mm]’	1-2, 10, 21	3380	2315	–	–
23	Opt.	‘4/4 A Wall Disp. [mm]’	1-2, 10, 21	665	2720	–	–
24	Opt.	‘4/4 B Wall Disp. [mm]’	1-2, 10, 21	1495	2720	–	–
25	Opt.	‘4/4 C Wall Disp. [mm]’	1-2, 10, 21	3380	2720	–	–
26	Pot./Opt.	‘1/2 Side A OOP Detachment [mm]’	1-2, 10, 21	220	1425	–	–
27	Pot./Opt.	‘4/4 Side A OOP Detachment [mm]’	1-2, 10, 21	220	2560	–	–
28	Pot./Opt.	‘1/2 Side C OOP Detachment [mm]’	1-2, 10, 21	3770	1425	–	–
29	Pot./Opt.	‘4/4 Side C OOP Detachment [mm]’	1-2, 10, 21	3770	2560	–	–
30	–	‘Inertial Force [kN]’	1-2, 10, 22	–	–	–	–
31	Acc.	‘1/4 A Wall Acc. [g]’	21–27	885	615	138	–
32	Acc.	‘1/4 C Wall Acc. [g]’	21–27	3380	615	69	–
33	Acc.	‘3/4 A Wall Acc. [g]’	21–27	885	2150	176	–
34	Acc.	‘3/4 C Wall Acc. [g]’	21–27	3380	2150	42	–
35	Opt.	‘1/8 A Wall Disp. [mm]’	1-2, 10, 21	665	450	–	–
36	Opt.	‘1/8 B Wall Disp. [mm]’	1-2, 10, 21	1495	450	–	–
37	Opt.	‘1/8 C Wall Disp. [mm]’	1-2, 10, 21	3380	450	–	–
38	Opt.	‘Side A Window Corner Disp. [mm]’	1-2, 10, 21	1660	530	–	–
39	Opt.	‘Side C Window Corner Disp. [mm]’	1-2, 10, 21	3125	530	–	–

**Table 4:** CL-000-RF data organisation.

Col.	Instr.	Description	Offline	Location		Associated Mass	
				X [mm]	Z [mm]	1^st^ [kg]	2^nd^ [kg]
1	–	‘Time [s]’	–	–	–	–	–
2	Acc.	‘Shake Table Acc. [g]’	–	–	–	–	–
3	Acc.	‘Foundation Acc. [g]’	–	–	–	445	141
4	Acc.	‘Frame Acc. [g]’	23	–	–	–	–
5	Acc.	‘Side A Ret. Wall Acc. [g]’	–	–	–	108	98
6	Acc.	‘Top Beam Acc. [g]’	All Tests	–	–	–	–
7	Acc.	‘Side C Ret. Wall Acc. [g]’	–	–	–	108	98
8	Acc.	‘1/4 B Wall Acc. [g]’	–	2065	755	319	455
9	Acc.	‘1/2 A Wall Acc. [g]’	–	650	1415	241	333
10	Acc.	‘1/2 B Wall Acc. [g]’	–	2065	1415	176	177
11	Acc.	‘1/2 C Wall Acc. [g]’	–	3265	1415	241	333
12	Acc.	‘4/4 B Wall Acc. [g]’	–	2065	2555	284	284
13	Pot.	‘Shake Table Disp. [mm]’	–	–	–	–	–
14	Opt.	‘1/4 A Wall Disp. [mm]’	1, 9, 19, 22	1195	755	–	–
15	WP/Opt.	‘1/4 B Wall Disp. [mm]’	1, 9, 19, 22	2065	755	–	–
16	Opt.	‘1/4 C Wall Disp. [mm]’	1, 9, 19, 22	2940	755	–	–
17	Opt.	‘1/2 A Wall Disp. [mm]’	1, 9, 19, 22	1195	1415	–	–
18	WP/Opt.	‘1/2 B Wall Disp. [mm]’	1, 9, 19, 22	2065	1415	–	–
19	Opt.	‘1/2 C Wall Disp. [mm]’	1, 9, 19, 22	2940	1415	–	–
20	Opt.	‘3/4 A Wall Disp. [mm]’	1, 9, 19, 22	1195	2075	–	–
21	WP/Opt.	‘3/4 B Wall Disp. [mm]’	1, 9, 19, 22	2065	2075	–	–
22	Opt.	‘3/4 C Wall Disp. [mm]’	1, 9, 19, 22	2940	2075	–	–
23	Opt.	‘4/4 A Wall Disp. [mm]’	1, 9, 19, 22	1195	2735	–	–
24	Pot.	‘4/4 B Wall Disp. [mm]’	1, 9, 19, 22	2065	2735	–	–
25	Opt.	‘4/4 C Wall Disp. [mm]’	1, 9, 19, 22	2940	2735	–	–
26	Pot./Opt.	‘1/2 Side A OOP Detachment [mm]’	1, 9, 19, 22	155	1535	–	–
27	Pot./Opt.	‘4/4 Side A OOP Detachment [mm]’	1, 9, 19, 22	3865	2555	–	–
28	Pot./Opt.	‘1/2 Side C OOP Detachment [mm]’	1, 9, 19, 22	155	1535	–	–
29	Pot./Opt.	‘4/4 Side C OOP Detachment [mm]’	1, 9, 19, 22	3865	2555	–	–
30	–	‘Inertial Force [kN]’	1, 9, 19, 22	–	–	–	–
31	–	–	All Tests	–	–	–	–
32	–	–	All Tests	–	–	–	–
33	Acc.	‘3/4 B Wall Acc. [g]’	–	2065	2075	257	257
34	–	–	All Tests	–	–	–	–
35	Opt.	‘1/8 A Wall Disp. [mm]’	1, 9, 19, 22	1195	395	–	–
36	Opt.	‘1/8 B Wall Disp. [mm]’	1, 9, 19, 22	2065	395	–	–
37	Opt.	‘1/8 C Wall Disp. [mm]’	1, 9, 19, 22	2940	395	–	–
38	–	–	All Tests	–	–	–	–
39	–	–	All Tests	–	–	–	–

**Table 5:** CAV-000-RF data organisation.

Col.	Instr.	Description	Offline	Location		Associated Mass
				X [mm]	Z [mm]	1^st^ [kg]
1	–	‘Time [s]’	–	–	–	–
2	Acc.	‘Shake Table Acc. [g]’	–	–	–	–
3	Acc.	‘Foundation Acc. [g]’	–	–	–	460 + 530
4	Acc.	‘Frame Acc. [g]’	–	–	–	–
5	Acc.	‘Side A CS Ret. Wall Acc. [g]’	–	–	–	128
6	Acc.	‘Top Beam Acc. [g]’	All Tests	–	–	–
7	Acc.	‘Side C CS Ret. Wall Acc. [g]’	–	–	–	128
8	Acc.	‘1/4 B CS Wall Acc. [g]’	–	2990	695	251
9	Acc.	‘1/2 A CS Wall Acc. [g]’	–	995	1340	255
10	Acc.	‘1/2 B CS Wall Acc. [g]’	–	2105	1340	126
11	Acc.	‘1/2 C CS Wall Acc. [g]’	–	2990	1340	242
12	Acc.	‘4/4 B CS Wall Acc. [g]’	–	2105	2640	223
13	Acc.	‘Shake Table Disp. [mm]’	–	–	–	–
14	WP	‘1/4 A CS Wall Disp. [mm]’	1, 9, 16	995	695	–
15	WP	‘1/4 B CS Wall Disp. [mm]’	1, 9, 16	2105	695	–
16	WP	‘1/4 C CS Wall Disp. [mm]’	1, 9, 16	2990	695	–
17	WP	‘1/2 A CS Wall Disp. [mm]’	1, 9, 16	995	1340	–
18	WP	‘1/2 B CS Wall Disp. [mm]’	1, 9, 16	2105	1340	–
19	WP	‘1/2 C CS Wall Disp. [mm]’	1, 9, 16	2990	1340	–
20	WP	‘3/4 A CS Wall Disp. [mm]’	1, 9, 16	995	2070	–
21	WP	‘3/4 B CS Wall Disp. [mm]’	1, 9, 16	2105	2070	–
22	WP	‘3/4 C CS Wall Disp. [mm]’	1, 9, 16	2990	2070	–
23	Pot.	‘4/4 A CS Wall Disp. [mm]’	All Tests	–	–	–
24	Pot.	‘4/4 B CS Wall Disp. [mm]’	1, 9, 16	1990	2720	
25	Pot.	‘4/4 C CS Wall Disp. [mm]’	All Tests	–	–	–
26	Pot.	‘1/2 Side A OOP Detachment [mm]’	1, 9, 16	220	1425	–
27	Pot.	‘4/4 Side A OOP Detachment [mm]’	1, 9, 16	220	2640	–
28	Pot.	‘1/2 Side C OOP Detachment [mm]’	1, 9, 16	3770	1425	–
29	Pot.	‘4/4 Side C OOP Detachment [mm]’	1, 9, 16	3770	2640	–
30	–	‘Inertial Force [kN]’	1, 9, 16	–	–	–
31	Acc.	‘3/4 B CS Wall Acc. [g]’	1, 9, 16	2105	2070	242
32	Acc.	‘Side A CL Ret. Wall Acc. [g]’	1, 9, 16	–	–	137
33	Acc.	‘Side C CL Ret. Wall Acc. [g]’	1, 9, 16	–	–	154
34	Acc.	‘1/4 B CL Wall Acc. [g]’	–	2175	695	307
35	Acc.	‘1/2 A CL Wall Acc. [g]’	–	1085	1415	286
36	Acc.	‘1/2 B CL Wall Acc. [g]’	–	2175	1415	149
37	Acc.	‘1/2 C CL Wall Acc. [g]’	–	3265	1415	300
38	Acc.	‘3/4 B CL Wall Acc. [g]’	–	2175	2075	271
39	Acc.	‘4/4 B CL Wall Acc. [g]’	–	2175	2735	240
40	Opt.	‘1/4 A CL Wall Disp. [mm]’	1, 9, 16	1085	695	–
41	Opt.	‘1/4 B CL Wall Disp. [mm]’	1, 9, 16	2175	695	–
42	Opt.	‘1/4 C CL Wall Disp. [mm]’	1, 9, 16	3265	695	–
43	Opt.	‘1/2 A CL Wall Disp. [mm]’	1, 9, 16	1085	1415	–
44	Opt.	‘1/2 B CL Wall Disp. [mm]’	1, 9, 16	2175	1415	–
45	Opt.	‘1/2 C CL Wall Disp. [mm]’	1, 9, 16	3265	1415	–
46	Opt.	‘3/4 A CL Wall Disp. [mm]’	1, 9, 16	1085	2075	–
47	Opt.	‘3/4 B CL Wall Disp. [mm]’	1, 9, 16	2175	2075	–
48	Opt.	‘3/4 C CL Wall Disp. [mm]’	1, 9, 16	3265	2075	–
49	Opt.	‘4/4 A CL Wall Disp. [mm]’	1, 9, 16	1085	2735	–
50	Opt.	‘4/4 B CL Wall Disp. [mm]’	1, 9, 16	2175	2735	–
51	Opt.	‘4/4 C CL Wall Disp. [mm]’	1, 9, 16	3265	2735	–
52	Pot.	‘4/4 B CL Wall Disp. [mm]’	1, 9, 16	1960	2735	–

**Fig. 1:**
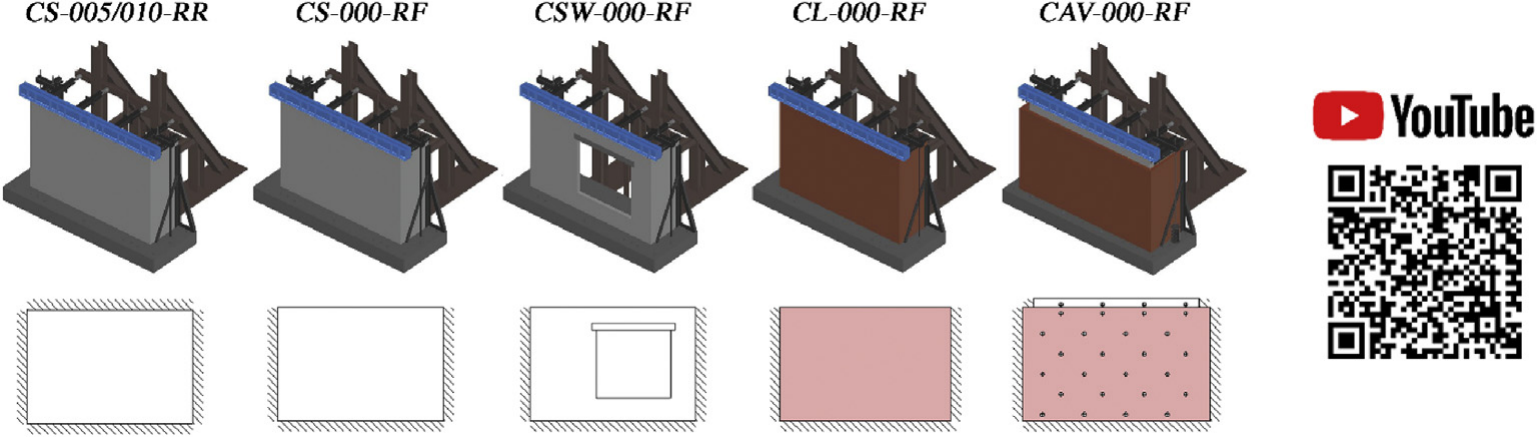
Schematic representation of the specimens and related boundary conditions. QR code to access YouTube playlist [2] documenting the failure of some specimens.

**Fig. 2:**
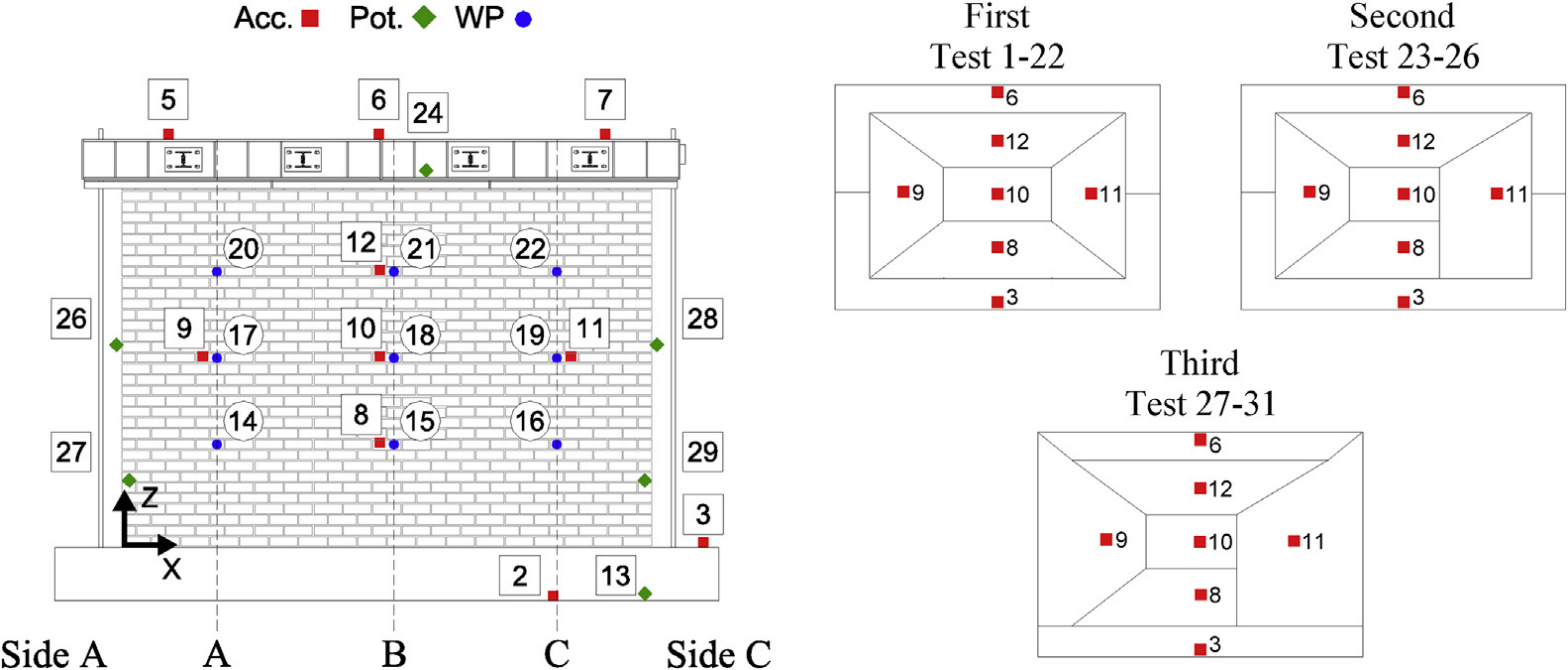
CS-010/005-RR Instrumentation scheme and mass distribution evolution with associated Test#.

**Fig. 3:**
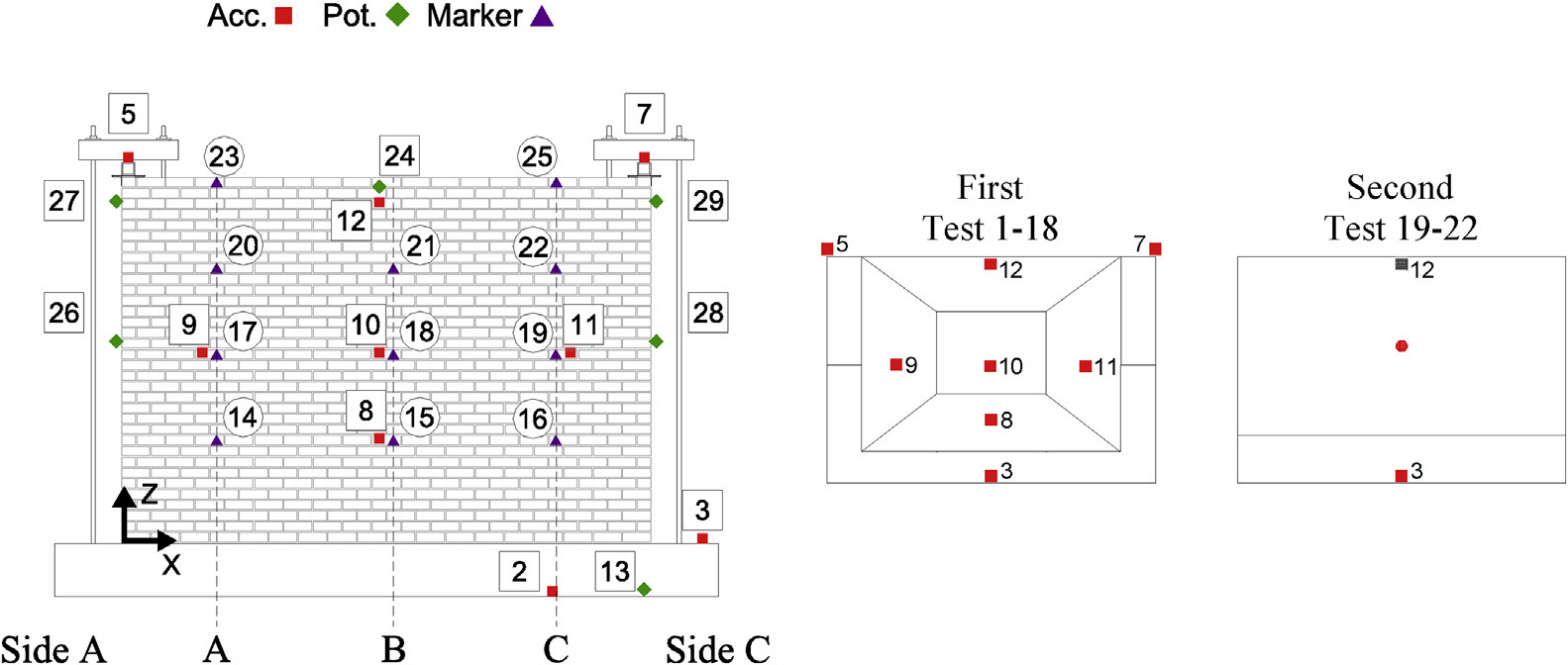
CS-000-RF instrumentation scheme and mass distribution evolution with associated Test#.

**Fig. 4:**
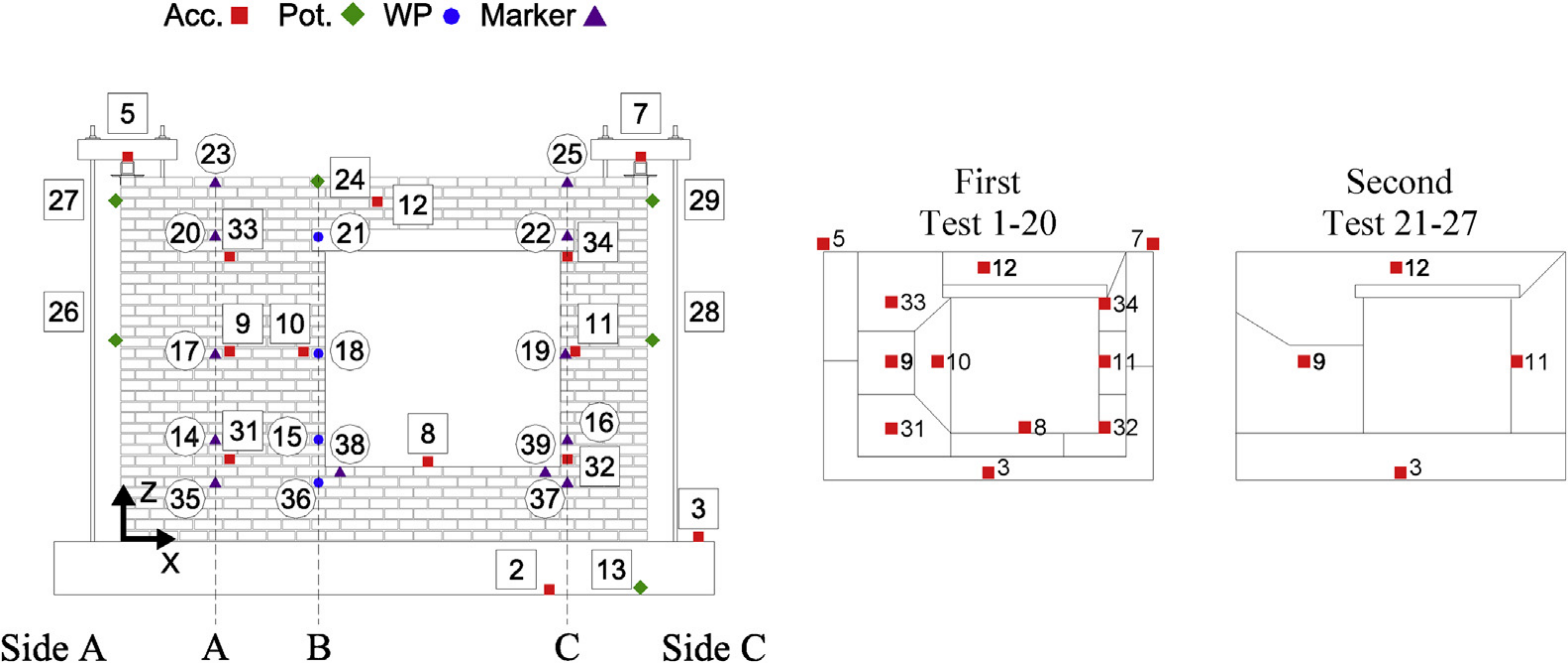
CSW-000-RF Instrumentation scheme and mass distribution evolution with associated Test#.

**Fig. 5:**
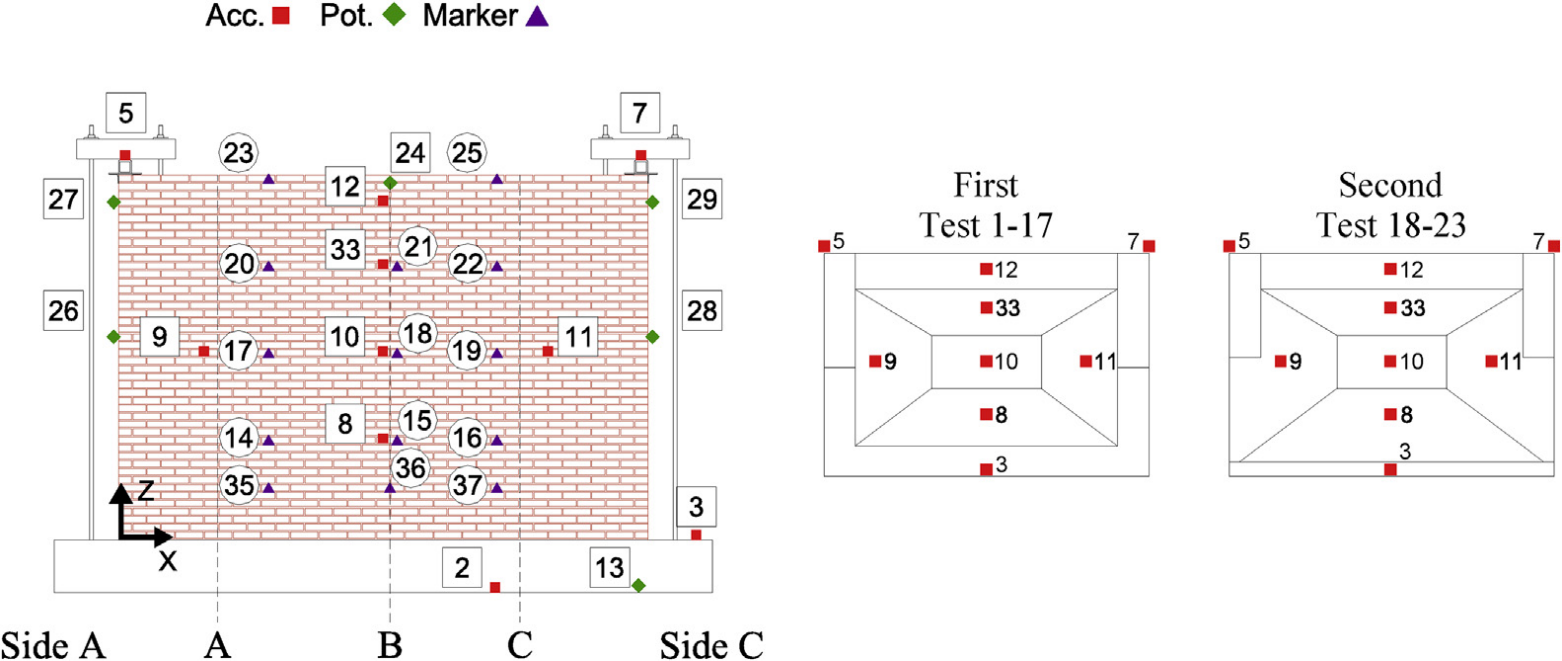
CL-000-RF Instrumentation scheme and mass distribution evolution with associated Test#.

**Fig. 6:**
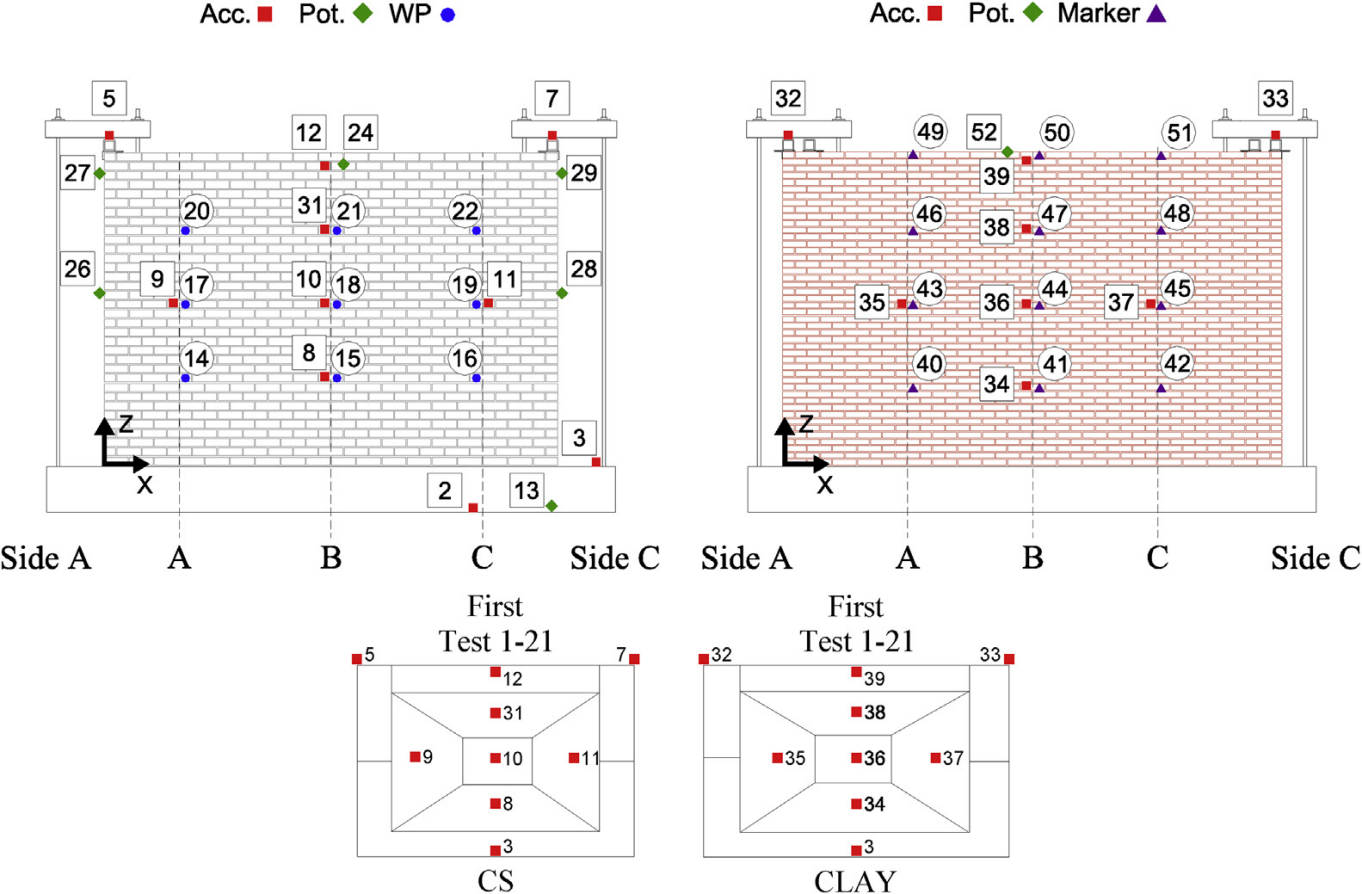
CAV-000-RF Instrumentation scheme and mass distribution evolution with associated Test#.

## 2 Experimental design, materials, and methods

This article presents the experimental data obtained from incremental dynamic testing of five full-scale URM walls subjected to two-way bending OOP seismic excitation. Four full scale URM walls (single leaf as well as cavity) were also previously tested by the authors under one-way bending excitation [3]. The data acquired from such tests may represent a benchmark for the development as well as calibration of numerical models to simulate the response of URM in the out-of-plane direction (*e.g.* Refs. [4], [5], [6]). This experimental data can also provide valuable insights on the comparison between results observed for walls with that of local two-way-bending failures observed in full-scale buildings (*e.g.* Tomassetti et al. [7]).

The five tested walls represent the first full-scale URM walls tested up to collapse that have been reported in literature. Three of these specimens were constructed in calcium silicate masonry (CS), one in clay (CL) masonry and another one was a cavity wall consisting of an inner leaf in CS and an outer leaf in CL masonry connected to each other by metal ties. All dynamic tests were carried out at the uniaxial shake table of EUCENTRE Pavia. The main input motions used in this part of the campaign corresponded to second floor accelerograms recorded either from a building prototype tested by Graziotti et al. [8] or from a calibrated numerical model of the tested building [5]. Low amplitude random excitations (RN) were used in between test runs to identify the dynamic properties of the specimens A summary of the naming adopted and boundary conditions associated with the tested specimens can be observed in Fig. 1. Characteristics of the employed input motions and their sequence along with the employed scaling factors are summarised in Table 4 of the reference article [1].

Every specimen was densely instrumented with sensors that recorded the dynamic response at various locations. The instrumentation adopted for each specimen consisted of accelerometers, potentiometers, wire potentiometers and a 3D optical acquisition system (used for all specimens except CS-005-RR). The location of all the instrumentation adopted for each specimen was decided based on the boundary conditions envisaged and correspondingly expected deformed shapes. Accelerometers were installed on the OOP panel of the specimen in order to record acceleration-time histories. Additional accelerometers were also installed at the specimen foundation, top beam, rigid frame and the return walls. Potentiometers were used to measure relative displacements associated with various locations of the specimen. Wire potentiometers attached to the rigid frame in several locations were used to record horizontal displacements relative to the shake table. Potentiometers were also adopted to record the relative displacements between the main panel and the return walls.

All data acquired was filtered from frequencies higher than 50 Hz. Accelerations and forces are provided in units of g and kN, respectively; displacements are given in mm. For each specimen, a folder is created named as the specimen: the folder containing data from all the tests corresponding to the second specimen is named as “CS-000-RF”. This folder contains .txt files for each test named as “TestT#” where “T#” refer to the same quantity provided in the testing sequence included in the reference article [1] (Table 4). Within each .txt file, the first column corresponds to a time vector whereas all other columns correspond to instrument readings. The instrument recordings contained in different columns for each specimen as well as coordinates of their exact location are provided in Table 1, Table 2, Table 3, Table 4, Table 5. Fig. 2, Fig. 3, Fig. 4, Fig. 5, Fig. 6 shows graphically the employed instruments for each specimen. In these tables and figures, Acc.: refers to accelerometer, WP: refers to wire potentiometer, Pot.: refers to potentiometer and Opt./Marker: refers to optical acquisition. Please note that moving towards higher intensities of shaking WP measurements were replaced with those obtained from a 3D optical (Opt./Marker) acquisition system.

Table 1, Table 2, Table 3, Table 4, Table 5 indicate also the mass associated with each accelerometer for the calculation of the inertial force of the OOP panel (provided in the *.txt* files). This associated lumped mass distribution changed throughout the testing sequence with the development of cracks and the adopted distribution throughout the testing sequence can also be found in Fig. 2, Fig. 3, Fig. 4, Fig. 5, Fig. 6. More details about how the inertial force was calculated can be found in the reference article [1]. It is worth noticing as in the case of CS-000-RF specimen during the last stages of testing (Test 19–22), due to lower number of instruments recording, a linear acceleration amplification was assumed along its height and half of the relative acceleration recorded by accelerometer 12 (marked in grey in Fig. 3) was assigned to the centre of the cracked panel. This was done in order to not overestimate the inertial force associated with the specimen. Additionally, the column “Offline” in Table 1, Table 2, Table 3, Table 4, Table 5 indicates test numbers (Table 4 of reference article [1]) when a particular instrument was not recording.

As an illustrative example, with reference to Table 2, column 3 of the file “Test6.txt” in the folder “CS-000-RF” corresponds to recordings of the 'Foundation Acc.’ when specimen CS-000-RF was subjected to FEQ2-DS3 scaled at 50% i.e. T#6 in Table 4 of the reference article [1].
