# Supplementary material for: Dataset from dynamic shake-table testing of five full-scale single leaf and cavity URM walls subjected to out-of-plane two-way bending
Source: Data Brief. 2019 Mar 20;24:103854. doi: 10.1016/j.dib.2019.103854 (PMC6480314; doi:10.1016/j.dib.2019.103854)
Supplement: Supplementary file 1 — Multimedia component 1 [file mmc1.pdf]

Conflicts of Interest Statement

Manuscript title: DATASET FROM DYNAMIC SHAKE-TABLE TESTING OF FIVE FULL SCALE SINGLE LEAF AND CAVITY URM WALLS SUBJECTED TO OUT-OF-PLANE TWO-WAY BENDING

The authors whose names are listed immediately below certify that they have NO affiliations with or involvement in any organization or entity with any financial interest (such as honoraria; educational grants; participation in speakers' bureaus; membership, employment, consultancies, stock ownership, or other equity interest; and expert testimony or patent-licensing arrangements), or non-financial interest (such as personal or professional relationships, affiliations, knowledge or beliefs) in the subject matter or materials discussed in this manuscript.

Author names: UMBERTO TOMASSETTI, LUCA GROTTOLI, SATYADHRIK SHARMA, FRANCESCO GRAZIOTTI

The authors whose names are listed immediately below report the following details of affiliation or involvement in an organization or entity with a financial or non-financial interest in the subject matter or materials discussed in this manuscript. Please specify the nature of the conflict on a separate sheet of paper if the space below is inadequate.

Author names:

This statement is signed by all the authors to indicate agreement that the above information is true and correct (*a photocopy of this form may be used if there are more than 10 authors*):

Author's name (typed)	Author's signature	Date
UMBERTO TOMASSETTI	[illegible]	05/03/2019
LUCA GROTTOLI	Luca Grottoli	05/03/2019
SATYADHRIK SHARMA	[illegible]	5th March 2019
FRANCESCO GRAZIOTTI	[illegible]	5/3/19
